# Cadmium and Cadmium/Zinc Ratios and Tobacco-Related Morbidities

**DOI:** 10.3390/ijerph14101154

**Published:** 2017-09-29

**Authors:** Patricia Richter, Obaid Faroon, R. Steven Pappas

**Affiliations:** 1 Tobacco and Volatiles Branch, National Center for Environmental Health, Centers for Disease Control and Prevention, 4770 Buford Hwy, MS F44, Atlanta, GA 30341, USA; PRichter@cdc.gov; 2 Agency for Toxic Substances and Disease Registry, Centers for Disease Control and Prevention, Atlanta, GA 30341, USA; OFaroon@cdc.gov

**Keywords:** tobacco, cigarette, smoking, chronic disease, toxic metals, cadmium, zinc, coronary disease, cardiovascular disease, COPD, pulmonary disease, cancer, prostate, pancreas, nephropathy

## Abstract

Metals are one of five major categories of carcinogenic or toxic constituents in tobacco and tobacco smoke. Cadmium is highly volatile and a higher percentage of the total tobacco cadmium content is efficiently transferred to mainstream tobacco smoke than many other toxic metals in tobacco. Inhaled cadmium bioaccumulates in the lungs and is distributed beyond the lungs to other tissues, with a total body biological half-life of one to two decades. Chronic cadmium exposure through tobacco use elevates blood and urine cadmium concentrations. Cadmium is a carcinogen, and an inducer of proinflammatory immune responses. Elevated exposure to cadmium is associated with reduced pulmonary function, obstructive lung disease, bronchogenic carcinoma, cardiovascular diseases including myocardial infarction, peripheral arterial disease, prostate cancer, cervical cancer, pancreatic cancer, and various oral pathologies. Cadmium and zinc have a toxicologically inverse relationship. Zinc is an essential element and is reportedly antagonistic to some manifestations of cadmium toxicity. This review summarizes associations between blood, urine, and tissue cadmium concentrations with emphasis on cadmium exposure due to tobacco use and several disease states. Available data about zinc and cadmium/zinc ratios and tobacco-related diseases is summarized from studies reporting smoking status. Collectively, data suggest that blood, urine, and tissue cadmium and cadmium/zinc ratios are often significantly different between smokers and nonsmokers and they are also different in smokers for several diseases and cancers. Additional biomonitoring data such as blood or serum and urine zinc and cadmium levels and cadmium/zinc ratios in smokers may provide further insight into the development and progression of diseases of the lung, cardiovascular system, and possibly other organs.

## 1. Introduction

Tobacco users are exposed to numerous toxic constituents, including toxic metals, from oral use of smokeless tobacco or inhalation of tobacco smoke [1,2]. Since neither the oral epithelium nor the pulmonary epithelium present a substantial barrier to many toxic substances [3,4,5,6], small particles and soluble toxic substances can be absorbed into epithelial and interstitial tissues and passed into the general circulation, resulting in exposure of other organs [7,8]. As a consequence of chronic exposure to tobacco smoke, select tissues may undergo toxicological changes resulting in pathological changes and tobacco-related morbidities.

Individuals exposed to elevated levels or repeated exposures to lower levels of toxins can often be distinguished from unexposed individuals by the presence of chemical compounds or their metabolites. However, these biomarkers of exposure sometimes do not adequately reflect biologically effective doses [9]. Biomarkers of exposure or potential harm that correlate with tobacco-related diseases or pre-pathological states inform health care providers and public health officials as they address individual and population level harms from tobacco use.

Many metals are readily quantified in organs or accessible biological matrices (e.g., urine and blood) and show utility as exposure biomarkers. In addition, elevated levels of some toxic metals have been linked with increased risk of adverse health outcomes [10]. Consequently, some metals are good candidates in select cases as biomarkers of exposure and potential harm. One constituent of tobacco and tobacco smoke, cadmium, is of considerable interest because it is toxic and because tobacco use is a major environmental source of cadmium exposure in the general population [11].

Strong evidence exists supporting cadmium as an inducer of proinflammatory cytokines, as a carcinogen, and as a substance that causes disease in many tissues in the body including the lungs and the kidneys [11]. Cadmium is classified as a Group 1 human carcinogen by the International Agency for Research on Cancer (IARC) [12]. IARC concluded that cadmium and cadmium compounds cause lung cancer and that positive associations exist between exposure to cadmium and kidney and prostate cancer [13]. The IARC report describes a potential mechanism of cadmium co-carcinogenicity that involves competitive zinc displacement by cadmium from several DNA repair enzymes. In addition to inhibiting DNA repair enzymes, cadmium exposure causes an inflammatory response by stimulating reactive oxygen species production by human polymorphonuclear leukocytes and phagocytic cells [13,14]. Chronic inflammatory response is implicated in carcinogenesis and in the development and progression of obstructive and fibrotic pulmonary diseases including COPD [15,16,17].

In stark contrast, dietary zinc is a beneficial and nutritionally essential element. Zinc was shown in animal studies to be antagonistic to cadmium tumorigenicity [12]. In humans, reduced zinc concentrations are associated with adverse health outcomes including impaired immune system function and a possible role in carcinogenesis [10,18,19]. An inverse relationship has been reported for serum cadmium and zinc levels amongst cigarette smokers [20]. Consequently, the ratio of cadmium/zinc may provide even more information as an indicator of increased disease risk than body burdens of either metal considered alone.

Few biomarkers have been validated as predictors of disease development [9]. This review examines cadmium and zinc levels in smokers and assesses associations between cadmium concentrations in blood, urine, and tissues with tobacco-related diseases. Available information for cadmium/zinc ratios is presented and contrasted with cadmium concentrations alone.

## 2. Origin of Toxic Metals in Tobacco and Transfer of Metals from Tobacco to Smoke

Tobacco plants absorb metal ions and compounds from the soil through their roots, and by translocation from roots to leaves. Factors that influence the levels of metals absorbed by tobacco plants include native metal concentrations in soil, use of soil amendments such as phosphate fertilizers, animal waste, or sewage sludge, and soil pH [21,22,23]. Tobacco contains numerous bioavailable toxic metals, including arsenic, barium, beryllium, cadmium, chromium, cobalt, lead, manganese, nickel, and uranium [1,24,25]. Seventy three metals have been identified in tobacco smoke [2,26].

Metal transfer rates from tobacco to tobacco smoke are influenced by the volatility of the metal and cigarette design [2,27,28]. Though present at lower concentrations in tobacco than some other metals [24], cadmium readily transfers to mainstream smoke in concentrations higher than most other metals due to the volatility of the transported form [2,27]. Tobacco filler from over 300 packs of cigarettes purchased in 20 areas around the world between 1982 and 1984 varied in cadmium concentration between 0.29 to 3.38 µg/g tobacco [29]. More recently, cadmium concentrations in tobacco filler were analyzed and found to vary from 2.0 to 5.4 µg/g tobacco in 78 varieties of cigarettes purchased in China [30], and from 1.0 to 1.7 µg/g tobacco in 50 cigarette varieties purchased in the U.S. in 2011 [24]. Cadmium concentrations in mainstream cigarette smoke generated under the International Organization for Standardization (ISO) smoking regimen [31] for 50 Philip Morris International varieties ranged from 1.6 to 101 ng/cigarette [32]. Counterfeit U.S. cigarettes had mainstream smoke cadmium levels (ISO 3308 smoking regimen) [31] that were much higher than legal products, ranging from 36 to 295 ng/cigarette [33]. Under an intense smoking machine regimen, mainstream smoke cadmium levels of legal U.S. and Canadian cigarettes ranged from 81 to 200 ng/cigarette [2,34].

Zinc levels in tobacco are not reported as frequently as cadmium, but one study reported a range spanning 16.8 to 30.5 µg/g tobacco in 12 U.S. cigarette varieties [35]. Though a significant portion of zinc is liberated from tobacco during combustion, zinc is less volatile than cadmium and does not pass beyond the filter into mainstream smoke as efficiently as cadmium [27,36]. Furthermore, the recommended daily intake for zinc is orders of magnitude higher than the levels of zinc in tobacco smoke [37]. The levels of zinc in tobacco smoke relative to physiological concentrations suggest that biological levels of zinc would be negligibly affected by the small amount transported in tobacco smoke.

## 3. Cadmium and Zinc as Markers of Exposure to Tobacco Smoke

Smoking is a major non-occupational source of exposure to cadmium [11]. Smokers have higher concentrations of cadmium in urine, blood, hair, and tissues than nonsmokers whose principle source of exposure is the diet [38,39,40,41,42]. Multiple reports have shown correlations between urine cadmium concentration and smoking status, including correlations with number of pack years smoked and age-dependent accumulation [38,39,40,41].

The biological half-life of cadmium based on creatinine-adjusted urine cadmium concentrations is 14.2 to 23.5 years [43] and cadmium concentrations in urine increase with age among smokers over a similar demographic of nonsmokers [40]. The National Report on Human Exposure to Environmental Chemicals from the Centers for Disease Control and Prevention (CDC) reported a mean urine cadmium concentration of 0.172 µg/L (0.199 µg/g creatinine) for non-smokers versus 0.308 µg/L (0.336 µg/g creatinine) for smokers in the U.S. population (not stratified by age) [44]. The 90th and 95th percentile urine cadmium concentrations for smokers age 50 and older in the CDC study were 1.85 µg/L (1.68 µg/g creatinine) and 3.50 µg/L (2.08 µg/g creatinine), respectively, which are comparable to urine cadmium concentrations reported for male adult current and former smokers (age 53 ± 7 years) with industrial exposure to cadmium [45]. It is important to note that the 95th percentile urine cadmium concentrations for U.S. smokers age 50 and older are more than three times the urine cadmium concentrations for age-matched non-smokers in the 95th percentile [44]. Using 1999–2004 data from the same National Health and Nutrition Examination Survey (NHANES), Richter and colleagues reported population level creatinine adjusted and unadjusted cadmium data for smokers and nonsmokers with or without exposure to secondhand smoke (SHS) [40]. Smokers, but not nonsmokers with SHS exposure, had higher adjusted and unadjusted geometric mean urine cadmium levels (0.34 μg/g creatinine and 0.37 μg/L, respectively) than nonsmokers without SHS exposure (0.21 μg/g creatinine and 0.20 μg/L, respectively) [40]. Across several studies females have higher blood and urine cadmium levels than men [40,41].

Whereas urine cadmium concentrations correlate with chronic exposure; blood or serum cadmium concentrations provide information on recent cadmium exposures [11,39,41]. Reported blood cadmium concentrations and smoking status indicate correlations with recent smoking and number of cigarettes smoked per day [38,41]. Results from the German Environmental Survey II [38] showed that the blood cadmium grand mean concentration increased significantly with increasing cigarettes smoked per day, and blood cadmium concentrations were 3 to 6 times higher for current smokers at all percentiles than for never smokers at equivalent percentiles. Adams and Newcomb determined that smoking in the previous 5 days was associated with a 55% higher geometric mean blood cadmium level but not with increased urinary cadmium [41].

In the Adams and Newcomb study both blood and urine cadmium concentrations declined subsequent to smoking cessation with the decline in urine cadmium larger for men than for women [41]. An approximately 25% decrease in urine cadmium concentrations was seen among women and 40% among men within a year of smoking cessation. Blood cadmium concentrations decreased by approximately 70% of the relative difference between smokers and nonsmokers for both sexes. Urine cadmium concentrations declined further in subsequent years, but did not reach levels comparable to never smokers.

Typical biomonitoring analyses don’t include zinc as often as cadmium. Zinc, an essential nutrient, is physiologically important as an antagonist to oxidative challenge [18,46]. Sparse data exist on zinc concentrations in tobacco smoke [47,48]. During combustion, less zinc transfers from tobacco to smoke (0.4–2.7%) than cadmium (7–22%) [26]. Fresquez et al. reported negligible zinc concentrations relative to cadmium in mainstream cigarette smoke particulate [27]. Like cadmium [40], physiological levels of zinc may be influenced by sex and age [49]. Buxaderas et al. reported significantly lower blood zinc levels in women (5852 ± 1229 μg/L) than men (6070 ± 1053 μg/L) [50]. Galan et al. also reported significantly lower serum zinc levels in women than men and found an age dependent decline in serum zinc in men but not women [49]. Other studies found that zinc concentrations in blood, serum, and tissues were lower in smokers than nonsmokers [19,51,52]. A study examining Turkish pack-a-day smokers versus control subjects (age 30 to 52 years, not stratified by age or pack years of exposure), revealed that smokers had a significantly lower mean serum zinc concentration of 14,150 µg/L, compared to 16,050 µg/L in control subjects [51]. Other studies reported no association between zinc levels and smoking status [50,53,54].

Metals exposure is not tobacco use specific, but outside of uncontrolled occupational exposure [55], elevated cadmium concentrations in the lungs and other organs and bioaccumulation in the lungs are presented as biomarkers of exposure amongst smokers. The temporal presence of metals in blood and urine makes these matrices easily accessible and informative for exposure biomonitoring [11].

## 4. Evidence for a Role for Cadmium and Zinc in Tobacco-Related Morbidities

Some metals that transfer from the tobacco filler to mainstream tobacco smoke, such as arsenic, cadmium, mercury, and lead, are naturally very toxic. Other metals present in mainstream smoke are known to accumulate in tissues and reach potentially toxic concentrations following long term tobacco use [17,56]. Many of the metals found in tobacco smoke have been shown in various data to cause diseases of various organs and or cancers. These metals include aluminum, arsenic, cadmium, chromium, copper, lead, mercury, nickel and others [10,57]. However, smoking is not always shown to be a major source of exposure to these metals and it is not possible to attribute an adverse health outcome from tobacco use to any one metal.

Ultrafine tobacco smoke particles containing metals may pass through the pulmonary interstitial tissue intact or gradually dissolve, enter the circulation, and distribute to other organs before elimination from the body [7,8,39,41,54,58,59,60]. Cadmium accumulates in the kidneys [11,61,62,63] where levels can exceed cadmium levels in the lungs by 30 to 60 fold [62,63]. Although the lungs are the first organ impacted by inhalation exposure, the kidney is the principal organ targeted by chronic exposure to cadmium, whether ingested or inhaled [11]. Evidence for cadmium as a driver of increased risk of kidney disease is strong. Nephrotoxicity and renal tubular damage occur when levels of total cadmium in the renal cortex reach between 50 and 300 μg/g wet weight [11] or when cadmium bound to the metal binding protein metallothionein in renal tubular cells exceeds 200 µg/g [61]. A population level study of tobacco smoke exposure and urinary metals reported that older smokers in the United States have cadmium levels high enough to suggest elevated risk for cadmium-related toxicities such as renal tubular toxicity [40]. Recent evidence suggests that chronically elevated renal cadmium concentrations may interfere with iron homeostasis as well [64].

Increased cadmium concentrations induce expression of metallothionein. Zinc homeostasis also involves metallothionein binding and induced expression. A proposed mechanism by which cigarette smoking disrupts zinc bioavailability is by nonselective metallothionein binding facilitated by adaptive cadmium induced metallothionein synthesis [10,65,66]. Conversely, because zinc and cadmium compete for the same binding targets, zinc administration can purportedly reduce the adverse effects of cadmium, possibly by zinc induction of metallothionein synthesis [67,68].

## 5. Cardiovascular Disease

Several studies found increased cadmium concentrations or increased cadmium/zinc ratios in patients with cardiovascular disease. Navas-Acien et al. correlated elevated urine cadmium concentrations and smoking status with peripheral artery disease using 1999–2000 data from NHANES [69]. Navas-Acien et al. also reported correlations between elevated blood cadmium and prevalence of peripheral artery disease among smokers [70]. Elevated plasma homocysteine levels are associated with peripheral artery disease. After adjusting for increased blood cadmium and lead associated with smoking, the odds ratio for homocysteine as a causative factor in peripheral artery disease decreased to less than 1. The authors suggested elevated blood cadmium and lead as possible causative agents for peripheral artery disease and possible causative or associative agents in the elevation of plasma homocysteine levels [71].

Using 1988–1994 data from NHANES, Everett and Frithsen reported a significantly higher odds ratio of 1.46 (95% CI 1.01–2.13) for myocardial infarction as “determined by electrocardiogram” with urine cadmium concentrations of 0.88 µg/g creatinine or greater, compared to the risk with urine cadmium concentrations of 0.43 µg/g creatinine or lower [72]. Current smokers in the study had urine cadmium concentrations greater than 0.88 µg/g creatinine. Of note, the association between increased urine cadmium and myocardial infarction persisted in never smokers with an odds ratio of 1.85 (95% CI 1.10–3.14) for never smokers with urine cadmium of 0.88 µg/g creatinine or greater compared to never smokers with urinary cadmium below 0.43 μg/g creatinine. Approximately 18% of never smokers in the cross-sectional data set had urine cadmium levels of 0.88 µg/g creatinine or greater, but cadmium concentrations were higher overall in former smokers than never smokers [39,72,73]. The odds ratio for myocardial infarction among never smokers with cadmium concentrations of 0.88 µg/g creatinine or greater was approximately equal to the odds ratio for smokers with the same urine cadmium concentration range [72]. This strengthens the evidence that cadmium is a key contributor to elevated risk of myocardial infarction and further suggests that elevated cadmium levels are a possible etiologic contributor, regardless of the source. A prospective cohort study of 3348 American Indian adults aged 45–74 years who had participated in the Strong Heart Study in 1989–1991 provided additional evidence that the known cardiovascular toxicity of cadmium might include contributions to cardiovascular disease. The group found that elevated urine cadmium concentration, considered as a biomarker of long-term cadmium exposure, was associated with increased incidence and mortality from cardiovascular disease [74]. The geometric mean cadmium level in the study population was 0.94 μg/g creatinine (95% confidence interval = 0.92–0.93), higher than the 0.88 urine μg/g creatinine cadmium concentration described by Everett and Frithsen as associated with increased risk of myocardial infarction [72]. They concluded that cadmium exposure is a risk factor for cardiovascular disease with 1084 cardiovascular events, including 400 deaths among 3348 study subjects [74].

In an early study of 47 male and female myocardial infarction patients Ponteva et al. reported higher mean blood cadmium levels and lower mean plasma zinc levels than in 37 healthy control subjects [75]. Patients with myocardial infarction and control subjects had similar smoking habits (percentages of nonsmokers, moderate smokers, and heavy smokers). Ponteva et al. also analyzed blood cadmium to plasma zinc ratios. They determined that, among the myocardial infarction patients, blood cadmium to plasma zinc ratios were outside the normal range for 43% of the patients, though there was an approximately 40% overlap of the blood cadmium to plasma zinc ratios between the myocardial infarction patients and the healthy control subjects. When retested approximately two years later the ratio was lower, leading the authors to propose that an elevated cadmium/zinc ratio was more relevant to the acute stages of myocardial infarction [75]. Their findings are promising but further work is required to characterize correlations between elevated cadmium/zinc ratios and heart disease across a range of tobacco use histories (pack years, cigarettes per day). Evaluation of cadmium/zinc ratios in at-risk patients with no clinical evidence of disease could help determine if an elevated ratio is a useful predictor of precipitant factors such as coronary artery disease leading to myocardial disease. Re-evaluation using blood cadmium to blood zinc ratios may also provide stronger and more consistent data since some metals in blood are predominantly cell-associated [14].

In a study of smoking and nonsmoking hypertensive patients, higher cadmium concentrations in hair, blood, and urine samples were observed among the smoking hypertensive patients than among nonsmoking hypertensive patients [52]. Lower zinc concentrations were observed in hair and blood from the smoking hypertensive patients than from nonsmoking controls. While the data are not prospective in nature, the findings are relevant because the hypertensive patients who had higher hair, urine, and blood cadmium concentrations were smokers and because others have used cross-sectional data to report a concentration-dependent relationship between blood cadmium concentrations and the prevalence of hypertension [76]. However, in the study of smoking and hypertension, Afridi et al. reported that urine zinc concentrations from smoking hypertensive patients were higher than from nonsmoking controls, the opposite of the cadmium/zinc relationship in the blood of smokers [52]. This could be due to greater intra-individual variability in urine excretion rates than plasma concentrations or competition with cadmium for cellular metallothionein resulting in increased excretion of free zinc in the urine. Taken together, these two studies suggest elevated blood or urine cadmium concentrations as possible markers of risk for hypertension. Further research is needed to compare cadmium/zinc ratios in the blood or serum of smokers with cadmium/zinc ratios in urine in consideration of possible higher rates of zinc excretion among smokers. It would also be important to include lead exposure as a possible covariate in studies of cardiovascular disease risk including risk of hypertension [11,71].

In a large study of cardiovascular disease risk factors among postmenopausal women in the United States, Lee et al. found that women with high dietary zinc intake were less likely to smoke [77]. Though they did not focus on association between smoking and cardiovascular health, they found no significant difference in relative cardiovascular disease risk between study participants whose diets were lower in zinc versus those whose diets had higher zinc levels when alcohol consumption was 9 g alcohol or less per day. However, cardiovascular disease mortality risk was decreased for those in the higher dietary zinc levels when alcohol consumption was higher than 10 g per day. Data from this study suggest a possible inverse correlation between dietary zinc status and risk of cardiovascular disease but alcohol consumption is an important covariate. In a study of 3575 adults in India, Singh et al. similarly reported a significant correlation between low dietary zinc intake and risk of coronary artery disease [78]. These data together with the cadmium/zinc ratio data from Ponteva et al. [75] and the data associating blood or urine cadmium concentrations with cardiovascular disease risk lend support to the value of investing cadmium/zinc ratios in studies of tobacco use and cardiovascular disease.

## 6. Pulmonary Disease

The lungs are one of the two principle target organs for toxicity effects resulting from inhalation exposure to cadmium [11]. Elemental cadmium is efficiently transferred to mainstream smoke from tobacco [2,28,33]. Consequently, it is expected that cadmium concentrations in cell-free bronchoalveolar lavage (BAL) fluid would be substantially higher for active smokers than nonsmokers without inhalation exposure to cadmium. Indeed, Sundblad et al. found this to be the case. Additionally, higher concentrations of mRNA for tumor necrosis factor (TNF)-α in BAL macrophages were positively correlated with BAL cadmium concentrations, as were proinflammatory cytokines interleukin-6 (IL-6), interleukin-8 (IL-8) and matrix metallopeptidase 9 (MMP-9, an extracellular matrix degrading protease thought to be involved in tissue remodeling during development and progression of chronic obstructive pulmonary disease) in cell-free sputum [79]. Higher concentrations of neutrophils and CD8^+^ T-lymphocytes (inflammatory cells) in blood were also positively correlated with lavage cadmium concentrations. Levels of IL-6 and TNF-α have been shown by others to be higher with more advanced COPD while markers of repair (e.g., MMP-9) were lower [80]. The finding of elevated MMP-9 in the Sundblad study [79] can be interpreted to suggest that patients were at an earlier stage of COPD. Profiles of early and late stage inflammatory markers compared with blood or urine cadmium levels or cadmium/zinc ratio data may indicate if cadmium or cadmium/zinc ratio are sufficiently sensitive and specific to tobacco use history to be predictive of COPD or to provide a quantifiable measure of disease state or severity.

Mannino et al. examined NHANES data and concluded that after adjusting for covariates such as age, pack years of smoking, and job category each one log increase in urine cadmium concentration was associated with significantly lower forced expiratory volumes (FEV) in smokers (−2.06%) and former smokers (−1.95%). No association of lung function and urine cadmium concentration in never smokers was found, suggesting that the correlation between urine cadmium concentration and FEV for smokers was a function of pulmonary exposure [81]. In the VA Normative Aging study, Lampe et al. reported even lower FEV (−7.56%) for each log unit increase in urine cadmium concentration [82]. Lampe et al. also noted that there was a graded statistically significant reduction in FEV/FVC (Forced Vital Capacity) across smoking status in association with urinary cadmium concentration [82], adding additional support to the interpretation that inhalation exposure is instrumental in the reduced lung function attributed to cadmium. They concluded that “…chronic cadmium exposure is associated with reduced pulmonary function, and cigarette smoking modifies this association.” [82].

Lin et al. correlated NHANES nutritional zinc intake and urine cadmium excretion data with risk of obstructive lung disease. They reported that obstructive lung disease was associated with low self-reported zinc intake regardless of smoking status. Lin et al. reported no significant correlation between self-reported zinc intake and smoking status, whereas they found that pack years of cigarette smoking and urine cadmium concentrations (Spearman correlation *p* < 0.001), as well as smoking status were significantly correlated with each other. Although urine cadmium concentration was significantly correlated with pack years smoking, there was a stronger correlation between urine cadmium concentration and obstructive lung disorder than smoking status, which was apparently the principal source of the elevated cadmium concentrations. Based on the ratio of zinc intake to cadmium excretion, they observed that higher zinc intake to urine cadmium concentration ratios were protective against obstructive lung disorder. The authors concluded that zinc has a moderating and protective effect in cadmium-related lung disease from tobacco use [83].

Hassan et al. reported correlations between lung tissue cadmium and manganese concentrations and Global Initiative for Chronic Obstructive Lung Disease (GOLD) criteria levels for COPD patients [54]. There were significantly higher manganese and cadmium concentrations in lung tissue from smokers with more advanced stages of disease (GOLD 4) than for smokers who were subclinically symptomatic or asymptomatic (GOLD 0). There were no significant difference in zinc levels in lung tissue between GOLD 0 and GOLD 4 participants. Since elevated cadmium concentrations in smokers’ lungs [56] are reflected in elevated blood cadmium concentrations of smokers [41,70], it is feasible that serum or blood cadmium/zinc ratios could serve as surrogates for lung tissue cadmium/zinc ratios in studies of COPD onset and progression.

Zinc concentrations decrease or do not significantly change due to smoking status while evidence exists that elevated pulmonary cadmium concentrations attributed to smoking are reflected in elevated blood cadmium concentrationsb [41,54]. Consequently, cadmium/zinc ratios merit consideration as markers of potential harm for cadmium-related or smoking-related pulmonary diseases where zinc concentrations may be suppressed. For example, Morgan reported significantly elevated mean serum cadmium concentrations and suppressed mean serum zinc concentrations as distinct characteristics of patients with bronchogenic carcinoma, a leading cause of death in the U.S. population associated with almost 85% of pulmonary cancer cases, and a disease commonly associated with smoking [84,85]. In the study of 47 male patients with exclusively bronchogenic carcinomas versus 50 control patients with other neoplastic diseases, the mean cadmium/zinc ratio in serum of the patients with bronchogenic cancer was 0.0328, more than twice the mean ratio of 0.0143 in serum from control cancer patients. Though bronchogenic carcinoma is considered to be predominantly a smoking-related disease, there was no information on smoking status in this early study [84]. Similarly, Davies et al. assessed plasma zinc concentrations in 81 patients with malignant disease. They reported that all 39 patients with carcinoma of the bronchus had significantly “below the lower limit” of the normal range (75 µg/100 mL) of plasma zinc levels and lower than in patients with other malignant diseases [86]. Voyatzoglou et al. also reported a significant correlation between decreased serum zinc levels and bronchogenic carcinoma among 25 patients with this malignant disease. They further reported that patients with bronchogenic carcinoma had elevated urinary zinc excretion compared to control subjects [87], as was reported by Afridi et al. for hypertensive patients who smoked [52]. Andrews noted that mean levels of plasma zinc decreased with longer tumor duration from 11.56 µmol/L (one to three months) to 10.13 (one to three years) [88] although interpretation of this finding is limited by possible differences in the amount of time before the patients sought medical care and diagnostic technologies common at the time of this early study. The findings of Strain et al [89] contrasted with those of Morgan, Davies, and Voyatzoglou [84,85,86,87]. Though the Strain et al. study had fewer subjects, they found no significant difference in serum zinc concentrations among 12 patients with bronchogenic carcinoma than for 10 control patients, similar to the findings later reported by Smith et al. for a study of over 100 patients with bronchogenic carcinoma [90]. Strain and colleagues, however, did discuss the association between high cadmium concentrations and many types of cancer. They proposed closer study of elemental ratios (e.g., such as cadmium/zinc or copper/zinc) when looking at the relationship between element deficiencies or excesses and disease [89]. Although findings from multiple studies support the hypothesis that there is a relationship between elevated blood cadmium and pulmonary disease, more data on cadmium/ zinc ratios from patients at various stages of the disease are needed to fully elucidate the relationships between blood cadmium and zinc concentrations and progression of COPD and carcinoma of the bronchus. It has been suggested that zinc deficiency and increased tobacco-related cadmium exposures could result in increased incidences of COPD in developing countries [91,92].

Data from Lin et al. above suggested that at the nutritional level, dietary zinc is antagonistic to cadmium pulmonary toxicity [83]. At the biochemical level, data supports an antagonistic role for pulmonary cadmium exposure and zinc homeostasis. Xu et al. have shown that following chronic treatment with cadmium, lung epithelial cells undergo decreased apoptosis by loss of expression for the zinc importer ZIP8 resulting in decreased cadmium and zinc accumulation in cells. Additionally, treated cells also exhibited increased secretion of vascular endothelial growth factor and macrophage inflammatory protein-3 alpha (MIP-3α) [93]. Both changes facilitate angiogenesis and cell migratory properties and are likely indicative of cell transformation to an early stage of cancer.

## 7. Prostate Disease

Anetor et al. reported significantly higher cadmium and lower zinc in serum from 55 Nigerian smokers than from 41 non-smokers [20]. The average cadmium/zinc ratio in serum from smokers was 4.5 times the average ratio in non-smoker serum. Anetor et al. hypothesized that through toxicologic mechanisms or disruption of physiologic functions, high cadmium/zinc ratios may signal increased risk of prostate cancer in smokers [20]. Application of the cadmium/zinc ratio could be even more informative in developing countries where dietary zinc deficiency has been reported to rank among the top ten leading causes of death [91]. It has been proposed that zinc deficiency together with increased cadmium exposures from increased tobacco use in developing countries could result in increased incidences of prostate cancer [20,92].

In support of assertions that elevated serum cadmium/zinc ratios could indicate increased risk of prostate cancer, Costello et al. provided a systematic review of the association between low prostate zinc concentrations and development and progression of prostate malignancies. They contrasted the high variability of zinc concentrations associated with benign prostatic hyperplasia (between 531 and 845 µg/g dry tissue and from 234 to 774 µg/g wet tissue) with the “strikingly consistent” significant decreases in malignant prostate tissue zinc concentrations [19]. In a summary of data from other published studies [93,94,95,96,97,98,99,100,101], Costello et al. reported that while normal prostate zinc concentrations varied between 517 and 540 µg/g dry tissue or from 125 to 348 µg/g in wet tissue, zinc concentrations in prostate cancer tissue were reduced to between 150 and 272 µg/g dry tissue (half or less than normal prostate zinc concentrations) and from 39 to 147 µg/g wet tissue [19]. Habib et al. stated that decreased prostate zinc concentrations generally preceded a reduction in the dihydrotestosterone/testosterone (DHT/T) ratio commonly observed during neoplastic transformation of prostate tissue. They concluded that decreased prostate tissue zinc concentration was a pattern that could serve as a predictive index for prostate cancer [98]. Further study is warranted, but decreased zinc could possibly be an alternative indicator since DHT/T ratios may reverse in cases such as castration-resistant prostate cancer [102].

In a prostate cancer study, Ogunlewe and Osegbe reported that the mean (11 µmol/L) and range of serum zinc concentrations were lower and the mean (24.2 µmol/L) and range of plasma cadmium concentrations were higher in patients with prostate cancer than in healthy men (14.9 µmol/L zinc, 15.2 µmol/L cadmium). They concluded that the molar ratios of cadmium/zinc in plasma of prostate cancer patients under their observation were elevated versus patients who did not have prostate cancer, and that plasma cadmium/zinc ratios could be used as a marker for prostate cancer [100].

## 8. Cervical Cancer

Balasubramaniyan et al. reported plasma copper, zinc, and cadmium analyses for 238 patients at stages 1 through 4 of carcinoma of the uterine cervix, a cancer associated with smoking [103,104]. Their emphasis was on serum copper/zinc ratios but their data showed that plasma zinc concentrations were significantly decreased in patients with stages 2, 3, and 4 carcinomas compared with the stage 1 group. Plasma cadmium levels were significantly higher for patients with stage 3 or 4 carcinomas than the stage 1 group. An interesting observation was that zinc levels increased and cadmium levels decreased after radiation treatment. The authors proposed that reduction of tumor mass may be responsible for renormalization of zinc and the other elements; however, authors did not discuss any changes in tobacco use after diagnosis or during treatment [103].

## 9. Pancreatic Disease

In 2004 the U.S. Surgeon General reported that smoking causes pancreatic cancer [105]. Smoking is the most commonly reported risk factor for pancreatic cancer, and a significant risk of pancreatic cancer exists with smoking duration and pack years smoked [106]. Pancreatic cancer is projected to become the second leading cause of cancer-related death in the United States by 2030 [107].

Kowal et al. reported overall mean wet pancreas tissue cadmium concentrations for citizens from two major cities in the United States. They reported that concentrations increased with age from 0.33 μg/g (ages 10–19) to 0.80 μg/g (ages 50–59). For nonsmokers of all ages, they reported mean pancreas tissue (wet) cadmium concentrations between 0.05 μg/g and 1.28 μg/g, compared to cadmium concentrations between 0.15 μg/g and 1.94 μg/g for smokers of all ages [73]. The increase in tissue cadmium concentration with smoking was statistically significant. Among the same participants, average urine cadmium and blood cadmium concentrations were significantly higher for smokers than nonsmokers.

Schwartz and Reis proposed cadmium as an etiologic agent in the development of pancreatic cancer. They described biochemical evidence that cadmium is one of the most potent trans differentiation-inducing agents known for the pancreas, that cadmium accumulates in the pancreas with age, that increased pancreatic cancer rates are associated with exposure in geographical regions with high cadmium pollution, that workers with high occupational exposure to cadmium have increased mortality from pancreatic cancer, and that the pancreas tissues of smokers have approximately twice the concentrations of cadmium as nonsmokers [108]. Exposure to cadmium resulted in increase of the trans-differentiation of pancreatic cells, synthesis of pancreatic DNA, and increased oncogene activation, therefore, it considered as a major factor for pancreatic cancer [11]. Collectively these factors are consistent with cadmium exposure as a risk factor for pancreatic cancer and for smoking as the most commonly associated risk factor for pancreatic cancer. Schwartz and Reis did not make a cadmium/zinc ratio connection with pancreatic cancer, though they did comment on a relation between cadmium and zinc based on evidence from zinc-dependent mechanisms for tumor induction suppression [108]. García-Esquinas et al. did not find statistical evidence that low to moderate exposure to cadmium was a significant risk factor for several specific types of cancer in a Johns Hopkins analysis of data from the Strong Heart study cohort. However, they did report that low and moderate cadmium exposures were associated with increased hazard ratios for total cancer mortality, all smoking-related cancers, and specifically with cancers of the lung and pancreas [109].

Downregulation of the zinc importer ZIP8 was discussed above as a toxicological consequence of chronic treatment of pulmonary epithelial cells with cadmium [93]. Costello et al. reported loss of the ZIP3 transporter in adenocarcinoma tissue sections accompanied by loss of intracellular zinc during well differentiated and progressing pancreatic malignancy. They further reported that treatment with zinc at physiological concentrations (2.5 µM) was cytotoxic to PANC-1 pancreatic cancer cells [110,111,112]. In contrast, a review of the intricate regulatory role of zinc in pancreas physiology and a study by Li et al. discussed the overexpression of ZIP4 in 94% of clinical pancreatic adenocarcinoma specimens compared with surrounding normal tissue. The positive and negative predictive values of ZIP4 for pancreatic ductal adenocarcinoma ranged from 76.1 to 83.9% and 69 to 94.4% based on tissue sampling method [113]. In addition, there was a significant correlation of tumor stage with percentage and immunohistochemical intensity of ZIP4 positive cells and of tumor differentiation and patient survival with ZIP4 intensity [114]. These data suggest that multiple zinc membrane transporters play a role in the intracellular concentrations of zinc during the development and progression of pancreatic cancer. Further study of zinc membrane transporters as possible markers of cadmium exposure and initiation and progression of pancreatic cancer is warranted.

## 10. Oral Pathologies

Kazi et al. reported elevated cadmium/zinc ratios in blood and hair samples from oral cancer patients from south Asia who had either smoked or used smokeless tobacco products [58,59]. These authors reported significantly higher average cadmium concentrations and lower zinc levels in whole blood and hair samples from cancer patients than from controls. Use of either form of tobacco led to higher cadmium levels in blood or hair. While not a pathological condition, Yaprak et al. reported significantly higher concentrations of cadmium, lead, arsenic, and manganese in supragingival dental calculus obtained from smokers than from non-smokers. The average cadmium concentration in dental calculus obtained from smokers was 17.4 times higher than the average concentration in dental calculus obtained from non-smokers [115]. The smoking group was not stratified on the basis of cigarettes smoked per day.

Evidence for the involvement of metals in oral pathologies is found in other studies. Katsuragi et al. provided support for involvement of cadmium and possibly other metals in oral pathologies. Among patients with advanced periodontitis they observed higher metallothionein levels and a higher metallothionein-positive cell ratio in the oral epithelial prickle cells of smokers compared with non-smokers [116]. Elevated metallothionein levels in superficial layers of the oral epithelium were also reported by Johann et al. in association with oral leukoplakia with moderate dysplasia [117]. Leukoplakia is a precancerous lesion for which smoking and oral tobacco use are the highest risk factors [118,119]; however there was no information on smoking status or tobacco use history for the patients providing tissue samples in the Johann study. Sundelin et al. reported elevated metallothionein levels in peripheral epithelial layers in association with squamous cell carcinoma of the tongue [118], a common oral carcinoma for which the primary risk factor is the use of tobacco products [115]. Metallothionein is typically immunochemically detected only in the basal cells in healthy normal oral epithelia. Expression of this protein in suprabasal and more superficial epithelia [116,117,120] strongly suggests that metallothionein synthesis in the oral epithelium is a cellular defense response against elevated oral toxic metal exposure [115]. It has been suggested that metallothionein-metal complexes can persist in the epithelial cells and permit transformed cell survival after tobacco-related pathological changes in oral epithelial tissue develop [121].

The data from Kazi et al. [58,59]. showing that oral cancer patients had who smoked or used smokeless tobacco products had elevated blood and hair cadmium/zinc ratios suggests, but does not prove a relationship between elevated blood or hair and cadmium or cadmium/zinc ratios and oral cancer. These data are limited as Kazi did not include oral tissue samples in their analysis and no information was provided on dietary zinc intake for the south Asian study participants. Although their focus was on correlation between copper/zinc ratios and oral pathologies, Jayadeep et al. reported that in a comparison between 92 patients with leukoplakia and oral squamous cell carcinoma and 45 age and sex-matched controls, serum zinc was significantly lower among male patients with leukoplakia and oral cancer [122]. The authors noted that malnutrition due to tumor location may have contributed to some nutritional deficiencies. Collectively the data suggest that extending these observations may provide evidence that supports the hypothesis that repeated tobacco use results in accumulation of cadmium and other metals in oral tissues and that cadmium or possibly a cadmium/zinc ratio is predictive of increased risk of oral cancer.

## 11. Conclusions

Tobacco smoke is a significant source of cadmium exposure, and tissue zinc concentrations in the body are affected by nutritional status and potentially by excessive cadmium intake. Smokers may have some nutritional deficiencies due to lower intake of fruits and vegetables [123]. Reduced body zinc levels may play a role in increased disease risk due to its antagonism of cadmium toxicity or there may be a more generalized homeostatic imbalance [124]. Imbalances that include elevated tissue, blood, or urine cadmium concentrations or decreased zinc concentrations are often associated with disease states (Table 1).

Some studies suggest that cadmium exposure may increase the risk of vascular and heart disease. Notably, two Johns Hopkins cross-sectional studies with study designs intended to represent the U.S. population based on analysis of NHANES data have shown correlations between blood and urine cadmium concentrations and peripheral arterial disease [69,70]. One study suggested a urine cadmium concentration that is associated with elevated risk of myocardial infarction in a large study of the U.S. population [72]. Data reported by Ponteva suggests that zinc status might have added value with regard to myocardial infarction risk relative to cadmium data alone and possibly uncover associations between smoking status, cadmium/zinc ratios, and risk of myocardial infarction [74].

Data have been presented as evidence that urine, serum, plasma, or blood zinc concentrations are negatively correlated with a number of diseases and with the progression of some diseases. As an example, Balasubramaniyan et al. described the inverse relation between copper/zinc ratios and the progression of cervical cancer [102]. Similarly, independent work by Habib, Costello, and others described a relationship between zinc concentrations and prostate cancer [19,97]. These data, together with reports from Anetor et al [20]. and Ogunlewe and Osegbe on association between elevated cadmium/zinc ratios in serum and plasma, respectively [99], and on smoking as well as prostate cancer suggest the possibility that blood cadmium/zinc ratios could inform efforts to develop predictors of prostate cancer risk. In particular, the correlation between elevated blood cadmium concentrations and cadmium/zinc ratios and prostate cancer risk [19,20] could inform risk assessment and potentially identify a tool for smoking interventions to mitigate disease risk.

Chronic exposure to cadmium causes pulmonary disease and lung cancer [11]. Elevated urine cadmium concentrations were shown to correlate with decreased FEV among smokers. Though only nutritional zinc intake questionnaire data were available to Lin et al. [82], these zinc intake data along with urine cadmium data were sufficient to establish a strong correlation between zinc intake, urine cadmium concentration, and to suggest cadmium inhalation from smoking as an etiologic factor in development of COPD. Interpretation of results from the Lin study would be strengthened by blood cadmium and zinc concentrations, considering that Hassan et al. were able to correlate lung tissue cadmium concentrations with GOLD level progression among COPD patients [53]. Taken together, the data of Morgan et al. [83], Davies et al. [85] and Strain et al. [88] on elevated serum cadmium and cadmium/zinc ratios correlated with bronchogenic carcinoma provide evidence of the directional relationship between these values and pulmonary health risks. Overall, correlations between cadmium inhalation and lung cancer as well as pulmonary disease suggest that further study is warranted to better establish relations between urine and blood cadmium concentrations, cadmium/zinc ratios, and stages of pulmonary disease. Other diseases for which there is evidence that smoking status and cadmium exposure play a role are pancreatic cancer and cancers of the lung [11,12,13,57,104].

Cadmium levels in urine, serum, plasma, and blood are significantly correlated with smoking status. In many cases, elevated cadmium levels are directly associated with disease states. Available information on zinc status as potentially antagonistic to cadmium toxicity were presented. Cadmium antagonizes or inhibits some of the necessary biochemical functions of zinc and supplemented zinc inhibits or reverses some toxicological effects of cadmium [113]. Consequently, the cadmium/zinc ratio may be more informative of health status than the concentrations of either metal alone. Additional research is needed to determine the sensitivity and specificity of cadmium and zinc concentrations in the same matrix, particularly blood, with the progression of disease state. Understanding the interplay between cadmium toxicity from tobacco and tobacco smoke exposure and zinc status may provide researchers and public health decision makers with a tool with more predictive ability than data for either cadmium or zinc alone.

Key information gaps exist for how cadmium/zinc ratios should be measured and interpreted. Additional research involving the quantitative analyses of blood or serum cadmium and zinc for determination of cadmium/zinc ratios could provide valuable insights for a range of applications including data mining by health researchers and both clinical and population level studies of tobacco use or other potential sources of cadmium exposure such as high occupations involving heavy metals.

Due to the interrelated processing and disposition of endogenous and exogenous elements, cadmium/zinc ratios warrant consideration as a marker for tobacco-related diseases. Blood cadmium/zinc ratios (in addition to plasma or urine) may reveal health-related changes as well as identification of early or late stage disease onset. In this review we present evidence for the possible utility of cadmium concentrations or cadmium/zinc ratios as indicators of exposure or increased disease risk. Cadmium/zinc ratios are promising tools for exploring the toxicological and pathological relationships between tobacco use and risk of various disease conditions.

## Figures and Tables

**Table 1:** Tobacco-related disease and evidence of cadmium and zinc involvement.

Target	Manifestation of Toxicity	References
Smoking	Elevated urine cadmium	[38,39,40,41,44,45,52,69,72,73,83]
	Elevated blood, serum, or plasma cadmium	[20,38,41,43,51,57,58,69,70,72]
	Lower blood, serum, or plasma zinc	[20,52]
	Elevated urine zinc	[52]
	Elevated blood, serum, or plasma cadmium/zinc ratio	[20,75]
	Elevated bronchoalveolar lavage cadmium	[79]
	Elevated lung tissue cadmium	[54,56]
	Elevated dental calculus cadmium	[115]
Cardiovascular Disease	Elevated urine cadmium	[74]
Peripheral artery disease	Elevated urine cadmium	[69]
	Elevated blood, serum, or plasma cadmium	[70,71]
	Elevated urine zinc	[72]
	Low dietary zinc	[78]
Myocardial infarction	Elevated blood, serum, or plasma cadmium	[75]
	Lower blood, serum, or plasma zinc	[75]
	Elevated blood, serum, or plasma cadmium/zinc ratio	[75]
Hypertension	Elevated urine cadmium	[52]
	Elevated blood, serum, or plasma cadmium	[52,76]
	Lower blood, serum, or plasma zinc	[52]
	Elevated urine zinc	[52]
Pulmonary Disease		
Inflammation markers	Elevated bronchoalveolar lavage cadmium	[79]
Reduced lung function	Elevated urine cadmium	[81,82]
Obstructive lung disease	Elevated lung tissue cadmium	[54]
	Elevated urine cadmium	[83]
	Low dietary zinc	[83]
Bronchogenic carcinoma	Elevated blood, serum, or plasma cadmium	[86]
	Lower blood, serum, or plasma zinc	[84,86,87]
	Elevated urine zinc	[85]
Prostate Disease	Elevated blood, serum, or plasma cadmium	[20,100]
	Lower blood, serum, or plasma zinc	[20,100]
	Elevated blood, serum, or plasma cadmium/zinc ratio	[20]
	Low tissue zinc	[19,94,95,96,97,98,99,100,101]
Cervical Cancer	Elevated blood, serum, or plasma cadmium	[103]
	Lower blood, serum, or plasma zinc	[103]
Pancreatic Cancer	Elevated tissue cadmium	[73,108,109]
Oral Pathologies	Elevated blood, serum, or plasma cadmium	[58,59]
Oral cancer/leukoplakia	Lower blood, serum, or plasma zinc	[58,59,122]
	Elevated blood, serum, or plasma cadmium/zinc ratio	[59,68]
